# Baseline-Free Damage Imaging of Composite Lap Joint via Parallel Array of Piezoelectric Sensors

**DOI:** 10.3390/s23229050

**Published:** 2023-11-08

**Authors:** Mohsen Barzegar, Artur L. Ribeiro, Dario J. Pasadas, Aadhik Asokkumar, Renaldas Raišutis, Helena G. Ramos

**Affiliations:** 1 Instituto de Telecomunicações, Instituto Superior Técnico, Universidade de Lisboa, 1049-001 Lisbon, Portugal; mohsenbarzegar.brz@gmail.com (M.B.);; 2 Prof. K. Baršauskas Ultrasound Research Institute, Kaunas University of Technology, 51423 Kaunas, Lithuaniarenaldas.raisutis@ktu.lt (R.R.)

**Keywords:** structural health monitoring, baseline-free, damage imaging, ultrasonic guided waves, sensor array, composite lap joint

## Abstract

This paper presents a baseline-free damage imaging technique using a parallel array of piezoelectric sensors and a control board that facilitates custom combinations of sensor selection. This technique incorporates an imaging algorithm that uses parallel beams for generation and reception of ultrasonic guided waves in a pitch–catch configuration. A baseline-free reconstruction algorithm for probabilistic inspection of defects (RAPID) algorithm is adopted. The proposed RAPID method replaces the conventional approach of using signal difference coefficients with the maximum signal envelope as a damage index, ensuring independence from baseline data. Additionally, conversely to the conventional RAPID algorithm which uses all possible sensor combinations, an innovative selection of combinations is proposed to mitigate attenuation effects. The proposed method is designed for the inspection of lap joints. Experimental measurements were carried out on a composite lap joint, which featured two dissimilar-sized disbonds positioned at the lap joint’s borderline. A 2D correlation coefficient was used to quantitatively determine the similarity between the obtained images and a reference image with correct defect shapes and locations. The results demonstrate the effectiveness of the proposed damage imaging method in detecting both defects. Additionally, parametric studies were conducted to illustrate how various parameters influence the accuracy of the obtained imaging results.

## 1. Introduction

Damage imaging techniques based on ultrasonic guided waves (UGWs) in structural health monitoring (SHM) applications primarily focus on defect detection and localization within structures, rather than providing detailed defect imaging. The primary reason for this lies in the fact that achieving higher-resolution imaging using UGWs requires the use of a densely populated grid of measurement points [1,2,3,4,5,6], which in the context of SHM, proves to be ineffective. Nevertheless, achieving high-accuracy tomographic results is crucial for the effective operation of health monitoring systems [7]. In the existing body of literature, SHM-based damage imaging methods frequently apply sparse piezoelectric sensor arrays [8,9,10,11,12]. Typically, a sparse array of piezoelectric transducers is either embedded within or mounted onto the structure, with one or several of these transducers acting as the excitation source of UGWs and the others acquiring the resulting propagated waves as signals.

In the context of SHM via visualization, various damage imaging algorithms have been introduced in the literature for the online assessment of structures. These include the delay-and-sum (DAS) imaging method [13,14,15,16], the time reversal method (TRM) [17,18,19], and the reconstruction algorithm for probabilistic inspection of defects (RAPID) method [20,21,22,23,24]. The DAS algorithm, originally adapted from radar technology, relies on exploiting residual signals and the group velocity of Lamb wave modes as they propagate. It involves shifting the residual signals from differed sensor combinations according to an appropriate time-shift definition and then averaging the signals. This method necessitates knowledge of Time of Flight (ToF), and therefore, it is most effective when a single Lamb wave mode is utilized for damage imaging [25]. Additionally, the conventional DAS algorithms require baseline subtraction [26,27,28]. The RAPID algorithm provides an alternative approach that does not depend on ToF information, and it is widely employed in UGW-based defect reconstruction algorithms. In the conventional RAPID algorithm, the damage index is determined using the signal difference coefficient, which is based on Pearson’s correlation coefficient between signals acquired with and without the presence of a defect. Therefore, the conventional RAPID method requires accurate baseline data, and its efficacy can be affected by environmental factors [29,30,31]. The time reversal method (TRM) based on UGWs is a candidate for baseline-free damage imaging. This method relies on the concept that dissimilarities between the original input signal and the reconstructed signal indicate the presence of damage. The reconstructed signal in this method is obtained through the use of a reversal in the time domain of the received signal. Due to factors such as dispersion behaviors of UGWs, similar to DAS, TRM also requires single-mode excitation for effective reconstruction. Additionally, in a study [32], baseline-free applicability limitations of this method were reported for metallic structures with structural discontinuities.

Given the reliance of DAS and TRM algorithms on single-mode propagation for efficient reconstruction, the RAPID algorithm emerges as a robust choice for applications that involve lap joint structures, particularly when UGW signals display dispersion and multi-modal behavior [33]. Nevertheless, in the case of composite lap joints, multiple factors influence the mechanical properties of these structures, ultimately affecting the propagation characteristics of UGWs within the structure. The dependency on environmental conditions makes the use of baseline data impractical. To address this challenge, this paper adapts the RAPID method for a baseline-free damage imaging technique. In this approach, rather than employing the typical signal difference coefficient utilized in conventional RAPID methods, the maximum envelope of each signal is considered as the damage index. Additionally, a parallel array of piezoelectric sensors is employed for sensor placement. Specifically, one line of the sensor array is affixed to the single composite plate, while the counterpart sensor array is attached to the bonded plate. The generation and reception of UGWs are achieved using parallel beams in a pitch–catch configuration. To effectively conduct customized combination of transmitters and receivers for the proposed damage imaging technique, a control board has been designed and experimentally implemented. Experimental measurements were conducted on a composite lap joint containing two dissimilar-sized artificially produced disbonds positioned at the borderline of the lap joint. In the determination of image similarity between acquired images and a reference image containing precise defect shapes and positions, a 2D correlation coefficient was employed. Moreover, a parametric study was carried out to investigate the impact of the parameters involved in the proposed damage imaging technique on the accuracy of the obtained imaging results.

The content of this paper is organized as follows: In Section 2, the composite lap joint specimen is presented along with the piezoelectric sensor array and the measurement set-up. Section 3 presents the proposed baseline-free damage imaging algorithm. Section 4 demonstrates the images obtained by the proposed methodology. In Section 5, the conclusion and future research directions are discussed.

## 2. Measurement Setup

**Figure 1:**
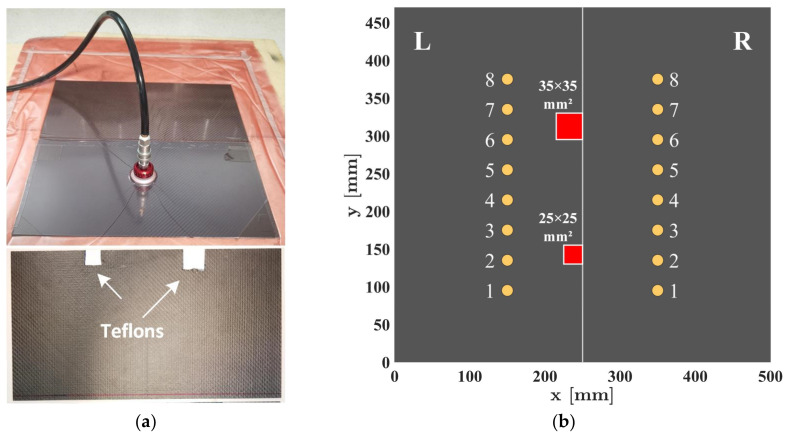
Composite Lap joint specimen: (**a**) vacuum bagging for fabrication and artificial disbonds attached to the borderline of one plate; (**b**) sizes, sensor positions, and disbond locations.

A SHM measurement system was developed to assess the condition of a composite lap joint containing two artificial disbonds of different sizes. These artificial disbonds were created by inserting Teflon films between the adhesive and one of the composite plates. To create the specimen, two 1 mm thick composite plates from Easy Composite (Stoke on Trent, UK) (product code: CFS-RI-1-0056) were bonded as a lap joint using Araldite 2011 adhesive. The vacuum bagging technique was employed to fabricate the specimen, and the disbonds were positioned at the lap joint’s boundary, as depicted in Figure 1a. Specimen sizes, sensor positions, and disbond locations are shown in Figure 1b, where a central borderline separates the adhesively bonded region (L) from the single plate side (R).

**Figure 2:**
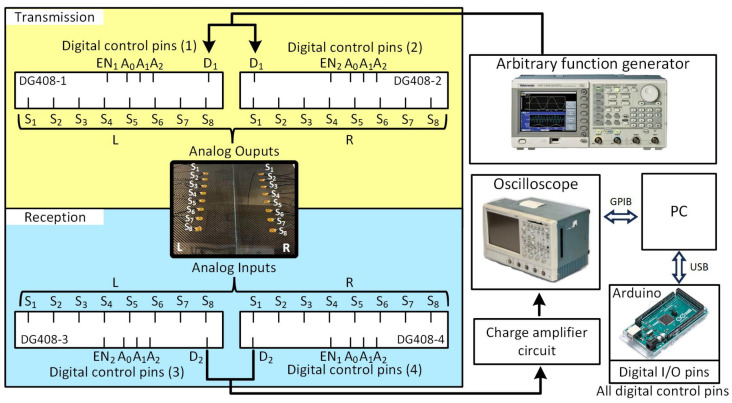
Schematic of the implemented measurement system.

The measurement system is equipped with a parallel array of piezoelectric sensors comprising 16 elements. Sensors used in this study were DuraAct piezoelectric transducers (P-876K025, PI Ceramics, Lederhose, Germany). These sensors are responsible for the generation and reception of UGW signals. Specifically, one set of eight sensors was attached to the individual composite plate, denoted as “R”, while another set of eight sensors was attached to the bonded region of the specimen, referred to as “L”. Both sets of sensors have the capability to function as both transmitters and receivers, facilitating the efficient collection of data required for the proposed damage imaging technique. The schematic representation of this implemented system can be observed in Figure 2.

**Figure 3:**
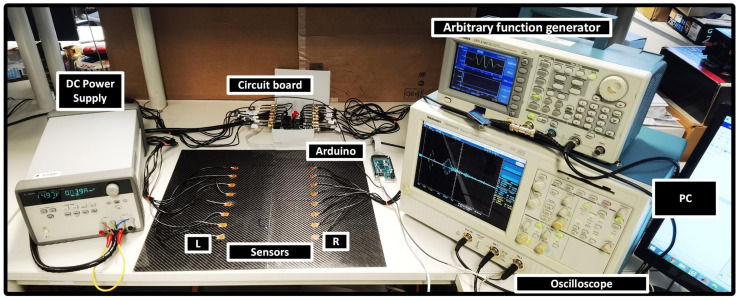
Experimental Setup.

To achieve a customized selection of sensor combinations for transmitters and receivers, a control system was devised by utilizing analog multiplexers/demultiplexers and an Arduino device. The control system includes four single-ended analog multiplexers/demultiplexers (DG408 from VISHAY, Malvern, PE, USA), each featuring eight channels. Among these, two of them were used to select the sensor, responsible for the excitation source (referred as DG408-1 or DG408 2), and the other two were used for the selection of the receivers (referred as DG408-3 or DG408 4). The switching of these multiplexers/demultiplexers was carried out through controlling 14 digital pins of the Arduino device. An oscilloscope (TDS5032B from Tektronix, Beaverton, OR, USA) was used to acquire the signal transmitted and received by the PZT sensor array. An arbitrary function generator (AFG3102 from Tektronix, Beaverton, OR, USA) was used as the excitation source to generate a tone burst with a five-cycle sinusoid waveform enveloped within a Hann window. For each customized selection of a transmitter, the output of the AFG was automatically routed to the PZT sensor responsible for the transmission of the wave to the plate. This routing was accomplished using either DG408-1 or DG408-2. Conversely, for each customized selection of a receiver, the output signal was directed to a charge amplifier via DG408-3 or DG408-4 to amplify the acquired signal prior to the acquisition by the oscilloscope. The pair of DG408-1/DG408-3 and DG408-2/DG408-4 share the same enable pins, respectively. Buffer circuits were introduced before the charge amplifier to ensure that the impedance mismatch between the sensor (which typically has a high output impedance) and the charge amplifier (which requires a low input impedance) is reduced. The customized selection of transmitters and receivers was carefully executed to acquire signals in parallel beams, based on the configuration required for the proposed damage imaging algorithm. In this way, each signal measurement involves the use of one transmitter and one receiver. Employing the switch, the next pair of sensors are activated. This process continued until the desired number of signals was acquired. The entire measurement system was under the control of a computer. The experimental configuration and setup are visually depicted in Figure 3.

**Figure 4:**
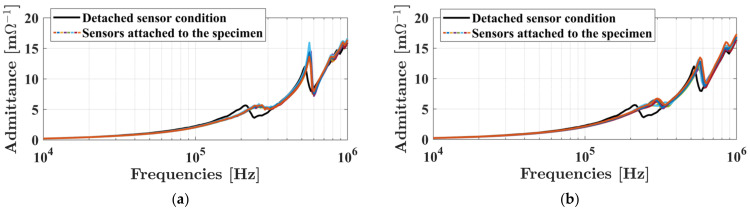
Magnitude of the admittance signature of the sensors including the sensor reference signature in air condition: (**a**) sensors attached above the single composite plate; (**b**) sensors attached above the bonded part.

Each of the piezoelectric sensors was attached to the specimen using epoxy adhesive. The quantity of epoxy adhesive and the quality of attachment can affect the sensors performance in propagating UGWs within the specimen. To assess the consistency of transmissibility between the sensors and the specimen post-bonding, the admittance signatures of all 16 sensors were measured using an LCR meter (3532-50 from Hioki, Nagano, Japan). In an effort to achieve uniform magnitude signatures for the range of frequency used in this study (200–300 kHz), some of the 16 sensors were intentionally detached and then re-attached to the specimen. The resultant signatures of the sensors, after these adjustments, are visually represented in Figure 4a,b for sensors attached to the single composite plate and bonded region, respectively. As a reference for a completely detached state, the admittance signature of the transducer without any attachment to the specimen is also illustrated in both figures as solid black curves. In a detached state, the transducers exhibit a resonance frequency of approximately 200 kHz. However, when they are connected to the specimen, their resonance frequency shifts to higher values (200–300 kHz).

## 3. Damage Imaging Algorithm

Probabilistic damage imaging in plate-like structures using a sparse array of piezoelectric transducers is commonly carried out through the generation and reception of UGWs and the subsequent analysis of recorded signals. Within this framework, the fundamental principle that forms the foundation for the conventional RAPID method, as one of the promising damage imaging techniques, involves comparing the pristine baseline condition to the current damaged state. This comparison is executed by pairing various combinations of two sensors, which generates a numerical value indicating the level of deviation between the baseline and current states. To quantitatively visualize this discrepancy, this numerical value is typically represented as a probability distribution over a scanning area. Conventionally, this distribution assumes an elliptical shape, plotted between the two sensors. The final damage imaging is usually obtained through the fusion of all images generated from different sensor pairings. The formulation of spatial damage intensity distribution $I_{ij}(x,y)$ in the RAPID method for the combination of two piezoelectric sensors (transmitter $S_{i}\left(x_{i},y_{i}\right)$, receiver $S_{j}(x_{j},y_{j})$) can be expressed as follows:

(1) $$I_{ij}\left(x,y\right)=DI\;\frac{\beta -R_{ij}(x,y)}{\beta -1},$$

where the elliptical shape distribution function is defined as follows:

(2) $$R_{ij}\left(x,y\right)=\left\{\begin{array}{cc} \frac{\sqrt{{\left(x-x_{i}\right)}^{2}+{\left(y-y_{i}\right)}^{2}}+\sqrt{{\left(x-x_{j}\right)}^{2}+{\left(y-y_{j}\right)}^{2}}}{\sqrt{{\left(x_{i}-x_{j}\right)}^{2}+{\left(y_{i}-y_{j}\right)}^{2}}} & R_{ij}\left(x,y\right)<\beta \\ \beta & R_{ij}\left(x,y\right)\geq \beta \end{array}\right.$$

In the equations presented above, β represents the scaling parameter that determines the extent of the elliptical distribution’s width. Conventionally, damage index DI is defined as the signal difference coefficient. However, in scenarios where baseline information is unavailable, the damage index can be alternatively defined as a feature extracted from the measured signal. In this study, the maximum amplitude of the signal, following the application of Hilbert transform ($\max\left|HT(s)\right|$), is considered as the damage index. In the conventional RAPID method, the final damage imaging distribution is obtained through a process of image fusion. This fusion involves combining all spatial damage intensity distributions according to the following equation:

(3) $${I_{tot}}^{conv}\left(x,y\right)=\sum_{i=1}^{N}\sum_{j=1,j\neq i}^{N}I_{ij}\left(x,y\right)$$

where ${I_{tot}}^{conv}\left(x,y\right)$ represents the conventional cumulative damage intensity distribution, and N signifies the total number of relevant sensors in the process. It should be noted that directly using $\max\left|HT(s)\right|$ as the damage index in Equation (1) and eventually in Equation (3) may not yield accurate damage imaging results unless all sensor combinations share identical distances and path conditions. This challenge is particularly accentuated for composite materials, since in these materials, defect influence on the signal tends to become obscured by different levels of signal attenuations in various pathways.

To address the challenges mentioned above, a parallel array of piezoelectric sensors is proposed with parallel beams for the generation and reception of UGWs. In this definition, the attenuation effect is avoided by grouping signals from waves that travel through identical pathways. UGWs within each of these groups encounter consistent effective properties, and any variation in amplitude of the signals indicates the presence of defects. In this way, all pathways that are identical are considered in a group and their DI values are modified through a normalization within their corresponding group. Subsequently, the final damage imaging result is obtained through accumulation of images obtained from each group. The proposed cumulative damage intensity distribution is conducted through the following equations:

(4) $${I_{tot}}^{prop}\left(x,y\right)=I_{0}+\sum_{k=1}^{M}\left(I_{k}^{DU}+I_{k}^{UD}\right),$$

(5) $$I_{0}=\sum_{i=1}^{N}\bar{{DI}_{0}}\cdot \frac{\beta -R_{i\left(j=i\right)}\left(x,y\right)}{\beta -1},$$

(6) $$I_{k}^{DU}=\sum_{i=1}^{N-k}\bar{{DI}_{k}}\cdot \frac{\beta -R_{i(j=i+k)}(x,y)}{\beta -1},$$

(7) $$I_{k}^{UD}=\sum_{i=1}^{N-k}\bar{{DI}_{k}}\cdot \frac{\beta -R_{\left(N-i+1)(j=N-k-i+1\right)}(x,y)}{\beta -1},$$

(8) $$\bar{DI}=\frac{\max\left(DI\right)-DI}{\max\left(DI\right)-\min\left(DI\right)},\;\;\bar{DI}=1\;if\;\;\max\left(DI\right)=\min\left(DI\right),$$

**Figure 5:**
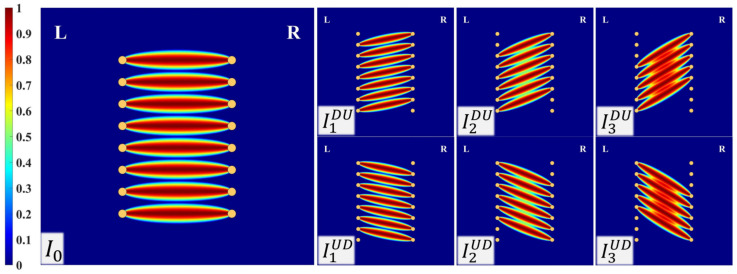
Cumulative damage intensity distributions through parallel beam emission and reception with a constant $\bar{DI}=1$.

In the above equations, $I_{0}$ is the damage intensity distribution obtained by parallel beams emitted from sensors within one array with their corresponding sensors located in the other parallel array. N is the total number of sensors in one side of the parallel array. $I_{k}^{DU}$ represents parallel beams oriented from the transmitters to receivers, which are positioned k sensors higher than those of the corresponding transmitters. Conversely, $I_{k}^{UD}$ represents parallel beams that are emitted from the transmitters to receivers located k sensors lower than the corresponding transmitters. M is the total number of oriented parallel beam groups considered for damage imaging. The value of M is constrained to fall within the range of 0 to N−1, allowing for a flexible selection based on the structure and sensor configuration. $\bar{DI}$ represents an array of damage indices derived from mapping the damage indices within each group of parallel beams onto a scale ranging from 0 to 1. Due to the lower energy of received UGWs that passes through a defect, $\bar{DI}$ formulation is designed in a way that the lowest DI value corresponds to 1, while the highest DI value corresponds to 0, and the reset of DIs receives in between accordingly. Note that the normalized vector of $\bar{{DI}_{k}}$ contains the damage indexes of both ${DI}_{k}^{UD}$ and ${DI}_{k}^{UD}$. Cumulative damage intensity distribution through parallel beam emission and reception are depicted in Figure 5 for β=1.02 and M=3, along with a constant $\bar{DI}=1$ for all sensor combinations.

## 4. Damage Imaging Results

**Figure 6:**
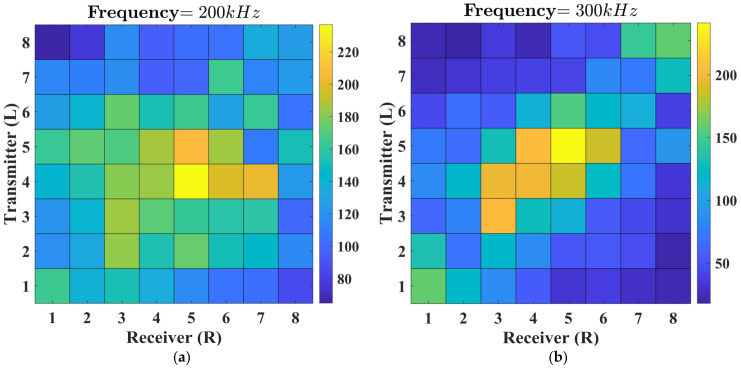
Heatmap of the maximum of the envelope for signals acquired by various sensor combinations and different central frequencies of the excitation: (**a**) 200 kHz; (**b**) 300 kHz.

Damage imaging results were obtained by analyzing the measured signals from various sensor combinations with different central frequencies of excitation. In Figure 6, the heatmap depicts the results of DIs, which was considered as $\max\left|HT(s)\right|$ of signals measured in millivolts (mV). Considering the heatmap results, increasing the excitation frequency leads to a more pronounced attenuation effect. The diagonal of the heatmap reflects the results obtained from parallel beams emitted by sensors in the transmitter array (L), aligned with their corresponding receiver sensors in the parallel array line (R). Consequently, under pristine conditions, it is expected to have the highest values along the diagonal, gradually diminishing by moving away from it. However, there are regions within the heatmap that do not exhibit this expected effect, indicating the presence of defects. This is clearer for the data obtained at the central frequency of 300 kHz, indicating that better damage imaging is expected for this frequency.

**Figure 7:**
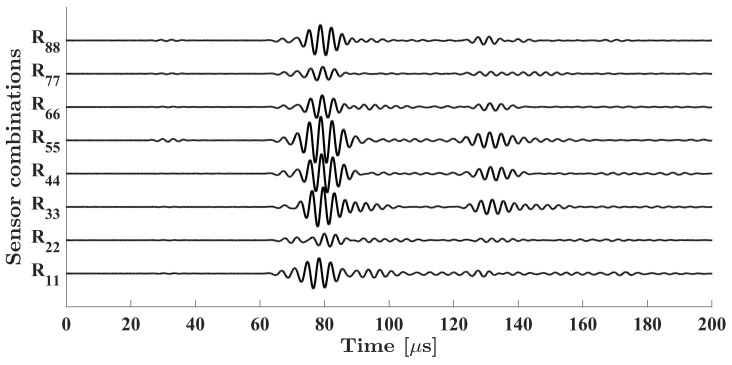
Example of signals acquired from sensor combinations within the group of $I_{0}$, excited with the central frequency of 300 kHz.

An example of signals acquired from sensor combinations in parallel beams within the group of $I_{0}$, where UGWs emitted from sensors within the array of transmitter (L) to their corresponding sensors located in the parallel array receiver (R) are plotted in Figure 7. These signals were obtained using the excitation of UGWs with the central frequency of 300 kHz. From this image, it is evident that the maximum amplitude of all signals occurred around the instant time of 80 µs. Upon closer examination around this time point, it can be seen that there are three signals (R_22_, R_66_, and R_77_) with decreased amplitudes compared to others. Among these signals, R_22_ is influenced by the presence of the smaller disbond, while R_66_ and R_77_ are affected by the larger disbond. Signal distortions induced by the presence of defects in sensor combinations without a direct path over defects tend to become more pronounced at later time intervals. However, the analysis of these time intervals is considerably complicated due to the occurrence of multiple refractions and reflections at the interface between the single plate and the bonded region and edges. Consequently, attempting to extract a damage index during these time periods remains exceedingly challenging. The heatmap data in conjunction with the sensor locations are used in the proposed damage imaging algorithm to represent the damage imaging results across the scanning area in a visual format. It is important to note that in real world applications, noise interference can affect SHM systems and electrical devices. Bandpass filters can be designed and applied to the measured signals to remove unwanted frequency components before supplying the signals to the proposed damage imaging algorithm.

**Figure 8:**
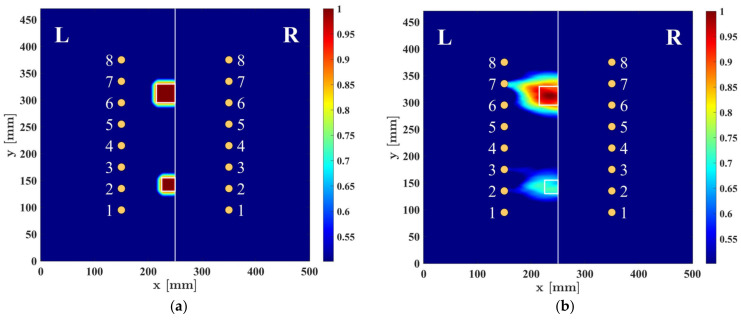
Damage imaging example: (**a**) reference image mask containing disbond amplitudes; (**b**) image obtained by the proposed method for the central frequency of 300 kHz.

The proposed damage imaging algorithm is employed for the detection of two disbonds with different sizes in the borderline of the composite lap joint. The spatial damage intensity distribution is considered to have the resolution of 1 × 1 mm^2^, which corresponds to the matrix size of 470 × 500 elements. In order to assess various parameters affecting the final damage imaging results, a reference mask matrix C was designed with same dimensions as the damage imaging matrix. This mask encompasses the shapes of the two defects depicted in Figure 8a, tapered by the Tukey function from all sides, except for the side at the borderline. Minor tapering was introduced because of the Teflon films not being perfectly square in shape and experiencing some stretching during insertion. The mask serves as a reference, and all damage imaging results are compared to it to determine a correlation coefficient denoted as ρ. It should be noted that the primary focus of this study is on the detection of delamination or disbonds in composite adhesive lap joints. Therefore, amplitudes of damage intensity distributions on the single plate (R side) are set to zero. The 2D correlation coefficient is computed using the following equation:

(9) $$\rho =\frac{\sum_{i}\sum_{j}\left[\left({I_{tot}}^{prop}\left(x_{i},y_{i}\right)-\mu_{{I_{tot}}^{prop}}\right)\left(C\left(x_{i},y_{i}\right)-\mu_{C}\right)\right]}{\sqrt{\left(\sum_{i}\sum_{j}{\left[{I_{tot}}^{prop}\left(x_{i},y_{i}\right)-\mu_{{I_{tot}}^{prop}}\right]}^{2}\right)\left(\sum_{i}\sum_{j}{\left[C\left(x_{i},y_{i}\right)-\mu_{C}\right]}^{2}\right)}}$$

In the above equation, $\mu_{{I_{tot}}^{prop}}$ and $\mu_{C}$ represent the mean values of all elements within the resulting damage intensity distribution matrix and the reference mask matrix, respectively. The value of the correlation coefficient varies between 0 and 1, with a higher value indicating a closer resemblance between the obtained final image and the reference mask image. Figure 8b shows an example of an image obtained from the proposed imaging algorithm using UGWs with the central frequency of 300 kHz. In this algorithm, the scaling parameter was considered as β = 1.05, and the number of oriented parallel beams was M=3. Also, the final image has been normalized and subjected to a threshold of −6 dB. In practical terms, this thresholding process involves setting all values that fall below 50 percent of the maximum amplitudes to the threshold value. This aids in enhancing the accuracy of the resulting image. The correlation coefficient for this case was obtained as ρ=0.754.

In the following subsections, the effect of three parameters on the final damage imaging result is studied. For this purpose, three different frequencies were considered for the central frequency of the excitation, including 200 kHz, 250 kHz, and 300 kHz. These values were selected as they are close enough to the resonance frequency of piezoelectric transducers. It should be noted that, in general, higher frequency waves propagate with smaller wavelengths, and UGWs with wavelengths as small as the size of the defect can ensure detectability. The range of frequencies for the excitation of UGWs in this study were ensured to interact with both defects of the lap joint specimen. Varying these frequencies, the effect of the scaling parameter and the number of oriented parallel beams were studied using the result of the correlation coefficient. In order to have a better observation of the impact of these parameters, the imaging results are zoomed in to the region bounded by the parallel sensor array, facilitating a clearer examination of the parameter study’s results.

### 4.1. Scaling Parameter

**Figure 9:**
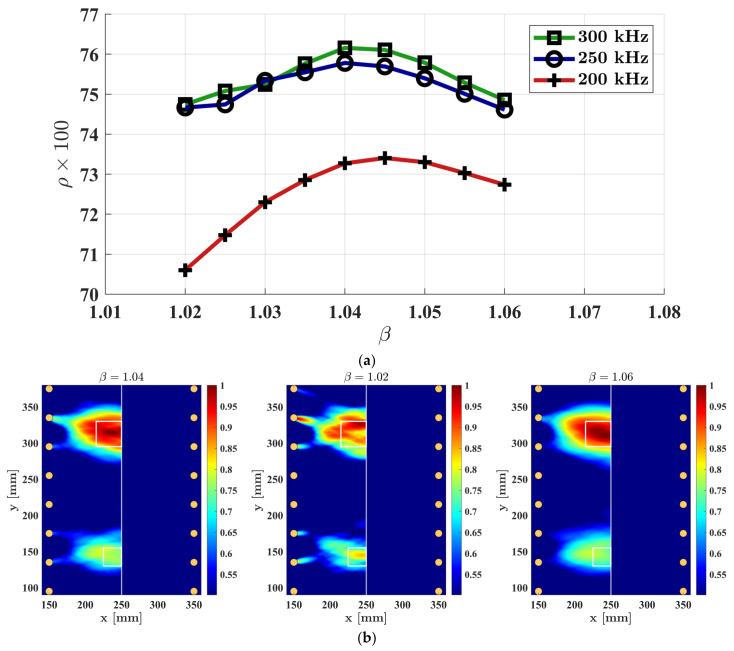
The effect of the scaling factor on the damage imaging result: (**a**) correlation coefficient obtained for various central frequencies of the excitation; (**b**) damage imaging using central frequency of 300 kHz.

The scaling parameter β controls the extent to which the elliptical probabilistic damage intensities are spread out. Depending on the size of the defect that the SHM system is targeting, this parameter can be adjusted. For the case of this study, the effect of the scaling parameter is investigated through increasing this value from β=1.02 to β=1.06, with an increment of 0.005. In this case, three different central frequencies of the excitation were considered, the number of oriented parallel beams was M=3, and the threshold value was set to −6 dB. Figure 9a shows the correlation coefficient obtained using different scaling parameter values. The results indicate a consistent trend across all frequencies where the correlation coefficient initially rises as the scaling factor increases, reaching its peak before subsequently declining. The optimal correlation coefficient values were achieved within the range of 1.04 to 1.05 for the scaling factor. Notably, by increasing the frequency from 200 kHz to 250 kHz, a significant change was observed, while between 250 kHz and 300 kHz, the results are very similar. Figure 9b illustrates three images obtained from using the optimum, the lowest, and the highest scaling parameter that was used in this experiment. For these damage imaging results, the excitation frequency was 300 kHz.

### 4.2. Number of Oriented Parallel Beams

**Figure 10:**
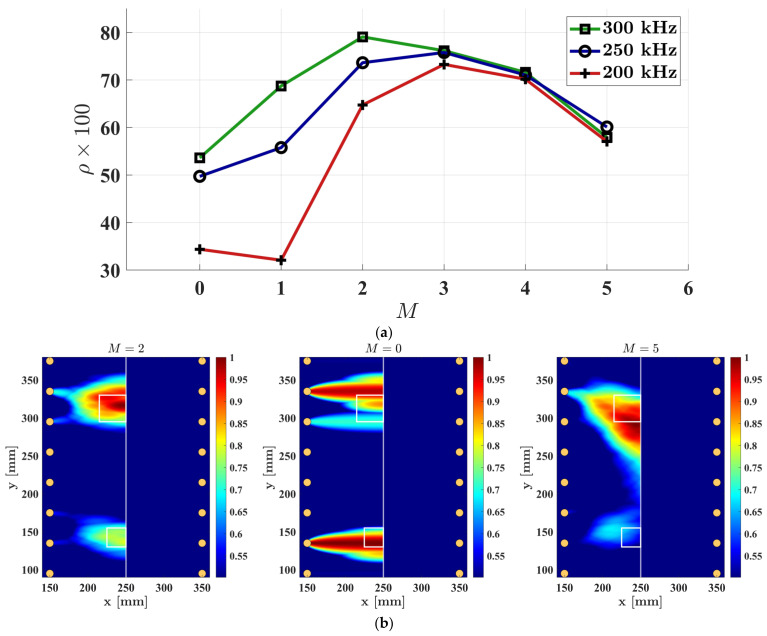
The effect of the total number of oriented parallel beams on the damage imaging result: (**a**) correlation coefficient obtained for various central frequencies of the excitation; (**b**) damage imaging using central frequency of 300 kHz.

In the case where no oriented parallel beams are used, the quality of imaging lacks resolution. Also, for a pair of parallel sensor arrays, increasing number of oriented parallel beams can skip extremist region as damage intensity distributions are concentrated to the center of the scan area and defects information will be buried or tend to show high amplitudes toward the center of the parallel array. To study this effect, various numbers of oriented parallel beams were considered from M=0, where no oriented parallel beams were used, to a condition where M=5. Similar to the previous parametric study, three excitation frequencies were employed to carry out the experiment. β=1.04 was considered for all damage imaging algorithms. The results of correlation coefficients for these frequencies, by varying the number of oriented parallel beams, are depicted in Figure 10a. In this figure, the same behavior is observed, which is an initial increase in correlation coefficient values, reaching an optimum point, followed by a decline. Additionally, for a lower frequency of the excitation, a higher number of oriented parallel beams was required to achieve effective damage imaging. However, in this specific structural and measurement condition, using M=4 and higher resulted in low accuracy for all excitation frequencies. Similarly, Figure 10b represents three images obtained using the optimal, lowest, and highest scaling parameters employed in this experiment. It is important to mention that these damage imaging results were obtained with an excitation frequency of 300 kHz.

Each of the parameters studied in this work have shown impacts on the final damage imaging results. An examination of the results in Figure 9a and Figure 10a reveals that, within the parameter range considered, the number of oriented parallel beams had a high impact on the obtained results. It was observed that employing either two or three parallel-oriented beams yielded the highest accuracy. When using an excitation frequency of 300 kHz, the scaling factor exhibited minimal effects. In contrast, with an excitation frequency of 200 kHz, the accuracy results generally reduced, and the sensitivity of accuracy to the scaling factor increased. Optimal correlation coefficient values for the scaling factor were found in the range of 1.04 to 1.05. Since the scaling parameter β governs the extent to which the elliptical probabilistic damage intensities are dispersed, it is evident that employing scaling factor values that are extremely small or large can lead to a decrease in accuracy.

## 5. Conclusions

Damage imaging for the visualization of defects has gained great recognition as a viable approach within SHM across various structures. In the existing literature, a predominant reliance on the baseline subtraction describes most UGW-based damage imaging techniques. However, the reliance on baseline data becomes impractical for composite adhesive lap joints due to uncertainties related to the mechanical and geometric properties of these structures. Therefore, this paper presented a baseline-free damage imaging method employing a parallel array of piezoelectric sensors, along with a control board that facilitates the customized selection of sensor combinations. This technique incorporates an imaging algorithm that uses parallel beams for UGW generation and reception in a pitch–catch configuration. To achieve this, a baseline-free RAPID method was adapted. In the proposed RAPID algorithm, the maximum envelope of each signal is considered as damage index, making it independent from baseline data. Furthermore, unlike the conventional RAPID method, which employs all possible sensor combinations, an alternative approach to sensor combination selection is presented. In the proposed method, the attenuation effect is circumvented by grouping signals from waves that propagate identical paths. Consequently, UGWs within each of these groups experience identical effective properties as they propagate along the same direction, and fluctuations in signal amplitude serve as indicators of defects. This method can be applied to metallic assemblies and other types of composite materials, as long as effective properties remain identical in each direction. Experimental measurements were performed on a composite lap joint containing two dissimilar-sized disbonds positioned at the boundary of the lap joint. Quantitative analysis of obtained images was conducted through the introduction of a 2D correlation coefficient in comparison to a reference damage image mask containing the real disbond shapes and locations. The effect of various parameters on the final damage imaging results was investigated. These parameters included the scaling factor, central frequency of excitation, and total number of oriented parallel beams. Future direction of this study will be on the investigation of various damage indexes, different damage intensity distribution shapes, and the presence of irregular-shaped disbonds on the accuracy of damage imaging.

## Data Availability

The data presented in this study are available on request from the corresponding author.
